# Lumbar Spinal Stenosis: The Reliability, Sensitivity and Specificity of the Nerve Root Sedimentation Sign

**DOI:** 10.5704/MOJ.1807.001

**Published:** 2018-07

**Authors:** MI Yusof, AF Azizan, MS Abdullah

**Affiliations:** Department of Orthopaedics, Universiti Sains Malaysia, Kubang Kerian, Malaysia; ^*^Department of Orthopaedics, Hospital Tunku Fauziah, Kangar, Malaysia; ^**^Department of Radiology, Universiti Sains Malaysia, Kubang Kerian, Malaysia

**Keywords:** lumbar stenosis, nerve root sedimentation sign, reliability, sensitivity, specificity

## Abstract

**Introduction:** This study is to evaluate the reliability, sensitivity and specificity of nerve root sedimentation sign (NRS) in our populations. The NRS is a radiological sign to diagnose lumbar spinal stenosis (LSS). It is claimed to be reliable with high sensitivity and specificity.

**Materials and Methods:** A total of 82 MRI images from 43 patients in Group A (LSS) and 39 patients in Group B (non LSS) were analysed and compared for the presence of the NRS sign. Two assessors were used to evaluate intra and inter-assessor reliability of this sign based on 56 (33 patients, Group A and 23 patients, Group B). The findings were statistically analysed using SPSS software.

**Results:** There was a significant association between spinal claudication and leg numbness with LSS (p<0.001 and Kappa=0.857, p<0.001). The inter-assessor reliability was also good (Kappa of 0.786, p<0.001).

**Conclusion:** The NRS sign has high sensitivity and specificity for diagnosing LSS. The sign also has good intra and inter-assessor reliability.

## Introduction

Lumbar spinal stenosis (LSS) is a condition when the spinal canal is narrowed and results in reduced space available for nerve elements. LSS is classified into central stenosis, lateral stenosis and combined central with lateral stenosis^1^ and occurs in up to 21% of those in retirement communities^2^.

The most common symptoms of LSS are intermittent neurogenic claudication, low back pain, paresthesia and subjective muscle weakness of the lower limbs. It is usually exacerbated by lumbar extension and improves with lumbar flexion^3^. Vascular claudication may resemble spinal stenosis, and some individuals experience unilateral or bilateral symptoms radiating down the legs rather than true claudication^4^.

The diagnosis of LSS typically relies on history, clinical examination and radiological investigation. Some patients can have a narrowed spinal canal without stenotic symptoms and do not require any treatment. Plain radiographs of the lumbar spine typically do not show spinal stenosis. The definitive diagnosis is established by either computerised tomography (CT) scanning or magnetic resonance imaging (MRI) with demonstration of reductions in the cross-sectional area of the central canal and neural foramina.

The nerve root sedimentation (NRS) sign is a new radiological sign first reported by Barz^5^. In the normal person during supine position, the nerve roots would sink posteriorly in the dural sac due to gravity. In those patients with LSS, the nerve root would not be able to sink but disperse ventrally. A positive NRS sign was defined as the absence of normally occurring sedimentation of the nerve roots in the dural sac. The NRS sign has been shown to discriminate well between patients with and without LSS and reported to be 94% sensitive and 100% specific^5^. However, the sign is not widely used probably due to its limited reliability study. In our practice, we treat a substantial number of patients with LSS. Having a new sign which is both diagnostic and prognostically reliable is useful in managing these patients.

This study is to assess the reliability, sensitivity and specificity in our patients with LSS and to compare them with studies reported by other authors.

## Materials And Methods

This study involved the evaluation of Magnetic Resonance Imaging (MRI) images of patients with and without degenerative lumbar spinal stenosis (LSS) who were randomly selected from our hospital patients’ registry from 1st January 2009 to 31st December 2013. They were divided into two groups: Group A those who were confirmed to have LSS and had spinal decompression surgery and Group B those who had no LSS confirmed by MRI performed for assessment of low back pain. Their clinical data were collected from the medical records for analysis. Patients with incomplete medical record, congenital spinal anomalies, peripheral arterial disease, diabetes mellitus with peripheral neuropathy, spinal deformity, infection or other musculoskeletal disorders with impaired walking ability were excluded from this study.

The method of evaluation followed the report by Barz^5^. The axial section of MR images which demonstrated the smallest cross-sectional diameter (CSD) were identified with the use of an electronic cursor at L1/2, L2/3, L3/4 and L4/5 levels. Then the axial images at the mid-vertebral body level above and below the levels demonstrated the smallest CSD were identified. Three axial images were then analysed for the presence or absence of the NRS sign [Philips Achieva 3.0 Tesla, Netherland].

**Fig. 1:**
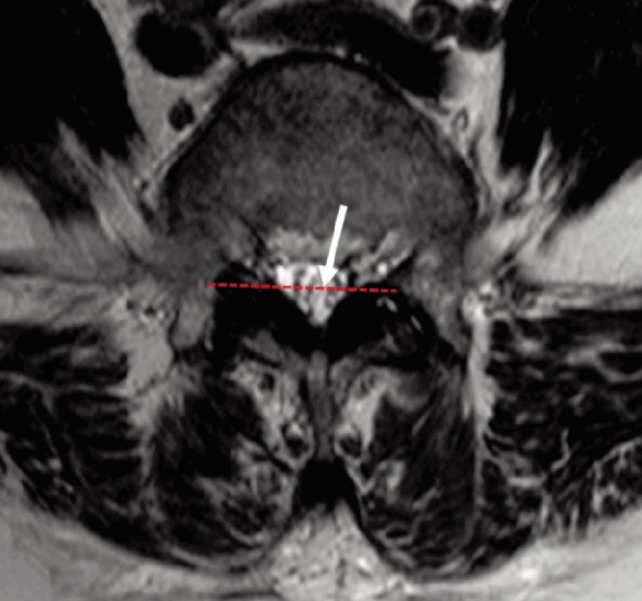
MRI T2 image of a patient with positive nerve root sedimentation sign. A positive sign was defined when most of the nerve roots (arrow) in the dura sac was located in the area above an imaginary line (dotted line) dividing the spinal canal into two halves.

**Fig. 2:**
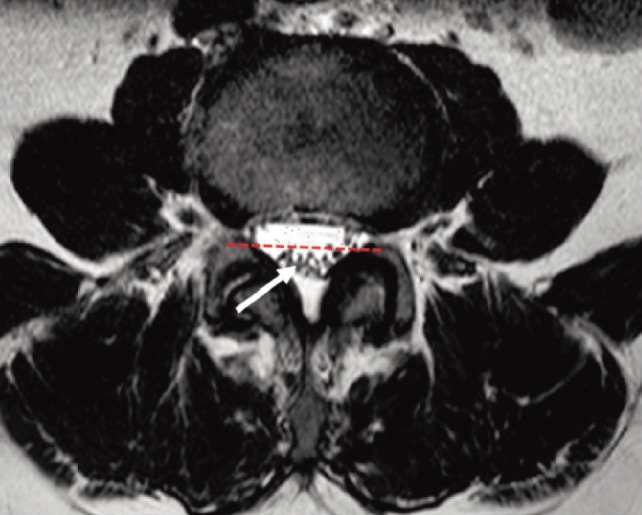
MRI T2 image of a patient with negative nerve root sedimentation sign. A negative sign was when the majority of the lumbar nerve root (arrow) was located in the dorsal half (dotted line) of the dural sac.

A positive sign was defined when most of the nerve roots in the dura sac were located in the area above an imaginary line dividing the spinal canal into two halves (Fig. 1). A negative sign was when the majority of the lumbar nerve root was located in the dorsal part of the dural sac^6^ (Fig. 2). The identification of the NRS sign was performed at the most stenotic level, one level above and one level below the affected level. The presence and absence of LSS were based on the clinical presentation and MRI assessment.

For intra and inter-assessor reliability assessment, the MRI images were coded and given to two assessors for evaluation of NRS sign independently. The observers were a radiologist and an orthopaedic surgeon. Both were blinded to the subjects’ clinical data. For intra-assessor reliability, the images were assessed by both assessors twice at two different occasions. The sequence of the MRI images was randomly changed for the second assessment to avoid bias.

All statistical analyses were conducted using IBM SPSS Statistics for Windows [version 22.0, Armonk, NY]. All variables were tabulated for descriptive statistics. All categorical variables were summarised in frequency (n) and percentage (%). The numerical variables were described in mean and standard deviation (SD) or median and interquartile range (IQR) depending on the normality of distribution. The t-test was used to test for differences in the distribution of numerical variables while the Pearson chi-square test was used categorical variables. The reliability, sensitivity and specificity of the NRS sign were analysed using chi-square test. The intra and inter-assessor reliability were analysed using the Kappa statistics. P-value of <0.05 was taken as significant. This study was approved by our institution human research ethics committee.

## Results

A total of 82 patients were enrolled in this study for analysis, consisting of 46 males and 36 females. Forty-three patients were in Group A and 39 patients in Group B. The mean age of Group A and B were 51.22 years (SD, 13.24) and 30.47 (SD, 9.85) respectively. Ethnic Malays made up the majority of the studied patients with half of them being government workers (Table I).

**Table I:** Socio-demographic characteristics of the patients in the study

Parameters	Frequency (%)	Mean (SD)
Age		43.12 (15.71)
Sex		
Male	46 (56.1)	
Female	36 (43.9)	
Race		
Malay	75 (91.5)	
Chinese	7 (8.5)	
Marital status		
Single	21 (25.6)	
Married	60 (73.2)	
Divorce/Widow	1 (1.2)	
Educational level		
None/Primary school	6 (7.3)	
Secondary school	35 (42.7)	
Tertiary education	41 (50.0)	
Occupation		
Housewife		18 (22.0)
Unemployed	10 (12.2)	
Self-employed		8 (9.8)
Government	42 (51.2)	
Private sector		4 (4.9)

The mean duration of symptoms in these patients was 3.4 years. All patients had low back pain, claudication symptom (39.0%), leg numbness (35.4%), leg pain (34.1%), buttock pain (30.5%) and thigh pain (12.2%). Motor weakness was present in 31.7% of patients and sensory deficit in 47.6%. Statistical analysis showed a significant association between clinical symptoms including spinal claudication, leg numbness, motor weakness and sensory deficit with LSS in Group A (p<0.001). Physical signs including leg weakness, buttock pain and thigh pain also showed significant association with LSS (p=0.037, 0.019 and 0.011 respectively) (Table II).

**Table II:** Clinical presentation of patients in Control and LSS Group (n=82)

Factors	Group Frequency (%) Non LSS n=39	LSS n=43	χ stat (df)	p-value^a^
Symptoms*				
Claudication				
No	39 (100.0)	11 (25.6)		
Yes	0 (0.0)	32 (74.4)	47.598 (1)	<0.001
Leg numbness				
No	39 (89.7)	18 (41.9)		
Yes	4 (10.3)	25 (58.1)	20.513 (1)	<0.001
Leg pain				
No	30 (76.9)	24 (55.8)		
Yes	9 (23.1)	19 (44.2)	4.053 (1)	0.044
Leg weakness				
No	38 (97.4)	36 (83.7)		
Yes	1 (2.6)	7 (16.3)	4.369 (1)	0.037
Buttock pain				
No	32 (82.1)	25 (58.1)		
Yes	7 (17.8)	18 (41.9)	5.518 (1)	0.019
Thigh pain				
No	38 (97.4)	34 (79.1)		
Yes	1 (2.6)	9 (20.9)	6.442 (1)	0.011
Physical examination* Rapidly relieved by sitting down or learning forward				
No	39 (100.0)	39 (90.7)		
Yes	0 (0.0)	4 (9.3)		0.118^b^
Sensory changes				
No	32 (82.1)	11 (25.6)		
Yes	7 (17.9)	32 (74.4)	26.149 (1)	<0.001
Reduce knee reflexes				
No	39 (100.0)	40 (93.0)		
Yes	0 (0.0)	3 (7.0)		0.243^b^
Reduce ankle reflexes				
No	39 (100.0)	34 (79.1)		
Yes	0 (0.0)	9 (20.9)		
Positive Barbinski’s sign				
No	39 (100.0)	41 (95.3)		
Yes	0 (0.0)	2 (4.7)		0.495^b^

* non mutually exclusive

a Pearson’s chi-square

b Fisher’s Exact Test

All 43/43 (100%) patients in Group A had positive NRS sign. In Group B, 7/39 patients (17.9%) had positive NRS sign. The sensitivity and specificity of NRS sign was 100% and 82.1% (95% CI= 70.0, 94.1).

MRI images of fifty-six patients were available for intra and inter-assessor reliability assessment, 33 in Group A and 23 in Group B. For Group A, Assessor 1 and 2 had rated positive NRS sign in 28/33 (84.8%; 95% CI= 72.6, 97.1) and 26/33 (78.8%; 95% CI= 64.8, 92.7) respectively. For Group B, both Assessor 1 and 2 had rated negative NRS sign in 22/23 (95.7%; 95% CI= 87.3, 100.0) patients and positive sign in 1/23 (4.3%) (Table III). Both Assessors 1 and 2 showed good intra-assessor reliability (Kappa= 0.785, p<0.001 and Kappa=0.857, p< 0.001). The inter-assessor reliability for two assessors was also good (Kappa=0.786, p< 0.001) (Table IV).

**Table III:** The sensitivity, specificity, PPV and NPV by Assessor 1 and 2 (n=56)

	Sensitivity %, (95% CI)	Specificity %, (95% CI)	PPV %, (95% CI)	NPV %, (95% CI)
Assessor 1	84.8	95.7	96.6	81.5
	(72.6, 97.1)	(87.3, 100.0)	(89.9, 100.0)	(66.8, 96.1)
Assessor 2	78.8	95.7	96.3	75.9
	(64.8, 92.7)	(87.3, 100.0)	(89.2, 100.0)	(60.3, 91.4)

**Table IV:** Intra and inter assessors reliability of the nerve root sedimentation sign (n= 56)

	Intra Assessor Reliability Kappa (95% CI)	Inter Assessor Reliability Kappa (95% CI)
Assessor 1	0.785 (0.62, 0.95)	0.786 (0.62, 0.95)
Assessor 2	0.857 (0.72, 0.99)	

## Discussion

In Group A, all patients were found to have positive NRS sign which indicated 100% sensitivity in diagnosing LSS patients with specificity of 82.1%. In Group B, negative NRS sign were found in 32/39 (82%) patients and only 7/39 (17.9%) patients were found to have positive NRS sign. The sensitivity of the NRS by Assessor 1 and 2 were 84.8% and 78.8% respectively, while the specificity by both observers was 95.7%. The sensitivity of the sign was higher in Assessor 1 probably because the assessor was a radiologist and more experienced in interpreting MRI images. Trivial abnormalities might be missed by the second assessor who was not a radiologist. However, the difference between both assessors was relatively small. It may suggest that the identification of the sign can be improved if the MRI images were seen by a radiologist, though a non-radiologist can also identify the sign satisfactorily. Training with a radiologist would improve identification of the sign. Nevertheless, the specificity of the signs was very high in both assessors.

The sensitivity of the sign in this study was comparable to the report by Barz^5^. Macedo *et al* in their study, found the NRS sign sensitivity of 54%. Its sensitivity increased to 82% when only severe cases as defined by Barz *et al* were included in the evaluation^7^. Another report also suggested that this sign demonstrated higher sensitivity and specificity in patients with severe morphological stenosis^8^. The NRS sign was found to be 100% positive in a group of patients with clinically diagnosed LSS with spinal claudication, walking distance of less than 200 meter and a dural sac CSA less than 80 mm^2^. Patients with nonspecific low back pain without claudication, walking capacity of more than 1000 m and a dural sac CSA more than 120 mm^2^, would have negative sign in 94%^5^. Thus, this sign could differentiate patients with LSS and without LSS accurately. However, as clearly mentioned by the authors, NRS sign by itself was not sufficient to confirm LSS.

The NRS sign was evaluated based on standard lumbar MRIs and can be easily identified. Positive NRS sign is a reliable sign to support the diagnosis of LSS with high sensitivity and specificity. The NRS sign has been proposed as a triage test to guide decisions about the further utilization of other existing tests. The sign is shown to be reliable to diagnose and to exclude LSS, thus becomes an additional diagnostic test for LSS. Other cumbersome techniques like treadmill test may then be omitted for diagnostic purposes for convenience and cost^9^.

Recently, Fazal *et al* reported a high sensitivity and specificity of the NRS sign in LSS patients requiring decompression surgery, indicating that the sign was also suggestive for significant lumbar stenosis which required surgery^10^. This finding was consistent with our study which showed all LSS patients who underwent surgical decompression had positive sign. NRS sign was consistently present in patients who had clinically significant lumbar stenosis and required surgery. NRS sign also can be used in postoperative follow-up assessment of the patients who had undergone surgical decompression, as reported by other authors^10,11^. There was also a report on the limited improvement of symptoms in non-surgically treated patients with positive sign^12^. These studies may indicate the prognostic value of NRS sign that require further investigations.

Narrow cross-sectional area (CSA) of the dural sac has been accepted as a good discriminator for the presence of LSS^13^. Both under and over-diagnoses of LSS are common using the CSA to quantify a stenosis. Presence of positive NRS sign and narrowed CSA would improve the specificity and accuracy of diagnosis and surgical decision for LSS. The NRS sign had good intra and inter-assessor reliability. Both assessors agreed on the positive NRS sign of most of the patients' MRI images.

Our observations were fairly similar to reports by others^6^ though lower than reports by Barz^5^. This study showed that there was significant association between spinal claudication and LSS with p-value of <0.001, comparable to Katz’s report which was the most common symptom associated with LSS^3^. Leg numbness, leg weakness, buttock pain and thigh pain also showed significant association with LSS with p-value <0.001, 0.037, 0.019 and 0.011 respectively. Presence of motor weakness and sensory deficit were also highly significant. Motor weakness and sensory deficits were seen in 58 and 52% of the patients respectively^14^.

There were a few limitations in this study. Firstly, the number of sample was quite small. However, the strength of this study based on the number of the patients was 86% and comparable with other studies. Secondly, all the patients enrolled in LSS group had undergone surgical intervention which indicated the severity of the condition. The NRS sign could be easily identified in this group of patients. It is ideal to have patients with different degrees of spinal stenosis and if the sign can be used to differentiate the severity of stenosis and decision for surgical treatment.

## Conclusion

There were significant association between spinal claudication, leg numbness, leg weakness, buttock, thigh pain and LSS. These clinical presentations were common and diagnostic for this condition. The NRS sign offers an additional assessment and prognostic tool for LSS and its treatment as it is highly sensitive, specific and reliable.

## Conflict Of Interest

The authors have no conflicts of interest to disclose.
